# Spike Dynamic and Epigenetic Malfunctions in Epilepsy: A Tale of Two Codes

**DOI:** 10.3389/fneur.2013.00063

**Published:** 2013-05-27

**Authors:** John Smythies, Lawrence Edelstein

**Affiliations:** ^1^Center for Brain and Cognition, University of California San DiegoSan Diego, CA, USA; ^2^Medimark CorporationDel Mar, CA, USA

## Introduction

The cellular processes that underlie the genesis and spread of epileptic seizures are currently poorly understood. This paper explores two hypotheses that seek to fill in some of these gaps. The first hypothesis relates to the role of the claustrum in the genesis of both complex partial and grand mal seizures in light of new and unique clinical data, and in modulating and integrating synchronized gamma oscillations in corticoclaustral circuits. The second hypothesis explores the possible role of epigenetic transcription factors, microRNAs, and exosomes in the kindling phenomenon. The conclusion is that the brain may possess two coding systems – one based on synchronized spikes for its software, the other on epigenetic processes that may modulate its dynamic hardware.

## Code 1: Synchronized Oscillations and Spike Timing

In a recent review, Zhang et al. (2011) state, “One of the most terrifying aspects of epilepsy is the sudden and apparently unpredictable transition of the brain into the pathological state of an epileptic seizure. The pathophysiology of the transition to seizure still remains mysterious.” Current ideas about the genesis of complex partial seizures are based on spreading cascades of interacting hypersynchronous oscillations between different functional networks. These are expressed in four recent reviews of this topic (Cavanna et al., 2011; Bertram, 2012; Blumfeld, 2012; Kramer and Cash, 2012). Particular attention is paid to the disruption of the network involving the thalamus and the frontoparietal association cortices that affects the level of awareness. Chipaux et al. (2013) have demonstrated with experiments in humans and rats that the disruption of conscious perception during an absence seizure is not due to an obliteration of information transfer in the thalamocortical system. The authors state, “Our findings thus dispute the widely accepted assumption that synchronized oscillations in thalamocortical loops disrupt conscious perception by filtering-out external sensory inputs and/or disallowing their allocation to the appropriate cortical assemblies.” Rather, they suggest that the loss of consciousness during absence seizures may be caused by a disruption of the normal information processing in large-scale brain networks. Stefan and Lopes da Silva (2013) argue that the concept of “local” and “generalized” epilepsies is now outmoded. They add that there is now compelling evidence, even in the case of Ideopathic Generalized Seizures and Childhood Absence Seizures, that seizures start in a well-defined brain area and spread at great speed to connected brain areas recruiting specific neuronal networks into typical oscillatory behavior. These accounts, however, consider only corticocortical and corticothalamic interactions; in none of these review papers is the claustrum even mentioned. Recent work, however, may throw some light on this mystery.

Koubeissi et al. (2012) have demonstrated that electrical stimulation of the claustrum, in a conscious patient with intractable complex partial epilepsy undergoing a preoperative investigation, produces a clinical state of immobility and complete lack of interaction with the environment, but retainment of some automatic movements, quite similar to cases of partial complex seizures. No such result was obtained when the nearby insula or white matter surrounding the claustrum was stimulated. Moreover, during the procedure, the EEG showed no evidence of ongoing seizure activity. This suggests that the stimulus had not simply triggered a seizure, and that the claustrum may somehow be involved in epileptogenesis. We therefore investigated how a recent hypothesis (Smythies et al., 2012), which suggests a specific functional role for the claustrum, might explain these observations.

This hypothesis states that the claustrum is involved in the integration of synchronized oscillations in the cerebral cortex. The mechanism suggested is as follows. Activation of two or more areas of the cortex will set up local areas of synchronized oscillations in those areas. Layer VI cortical neurons form feedback loops with selected claustral neurons according to the rule discovered by Pearson et al. (1982). This states that, if two cortical neurons (A and B) are paired by corticocortical connections, then the claustral neurons (C and D) that each cortical neuron projects (A–C and B–D) will themselves be anatomically connected. If other cortical neurons are not connected in this manner, than neither will the claustral neurons that they project to. In turn, the activated cortical neurons A and B will activate the interconnected claustral neurons C and D, which will then receive the synchronized oscillations. As claustral neurons are densely packed against one another, the interaction between C and D may be stronger than the interaction between A and B. In this way, the claustrum could amplify the synchronization. In an ever-changing environment, different corticoclaustral circuits would be activated sequentially, leading to competing intraclaustral oscillating systems. These would complete for dominance, with the winner activating the executive projection from the claustrum to the motor cortex, resulting in the appropriate behavior.

To adapt this hypothesis as a contribution to the explanation of epileptogenesis, we can develop the following scenario. Consider the situation where a local area of cortex develops, for any one of a number of reasons (Schevon et al., 2007), an epileptogenic focus. It is the tendency of such a focus to spread to local cortex where areas of local hypersynchrony will develop (Margineanu, 2010), further spreading to contralateral cortex via the corpus callosum to form a mirror focus, as well as to the claustrum. As a seizure develops, each of these foci will develop increasing excessive neuronal activity. In view of the compact nature of the claustrum, the effect of these excessive spikes may be magnified here also. At first, with only a few ictal pulses arriving, the claustrum might be able to maintain its normal function of integrating the synchronization of its inflow. However, as the ictal impulses steadily increase in number (Cymerbilt-Sabba and Schiller, 2012), a critical point would be reached in which the claustrum would no longer be able to maintain its normal function (because it was blocked by what is, in essence, excessive noise in the system), and a complex partial seizure would develop. This would be very similar to the effect produced by Koubeissi et al. (2012) using an electric current to disrupt claustral function. In this syndrome, other parts of the brain, which maintain such functions as standing posture and automatic movement, are still operative. However, should the ictal process continue, another threshold could be reached, at which point the seizure activity within the claustrum would trigger the activation of all claustral efferent neurons that project to nearly the entire cortex. This would send a massive number of spikes to the cortex, adding to the slower intercortical spread of ictal activity, thereby resulting in a grand mal seizure.

The similarity with “aclaustral rats,” following the intraclaustral injection of a specific toxin as described by Remedios (2012), is remarkable yet subtly different. The aclaustral rats could only make a few tentative, feeble, and abortive efforts to enter a wing of the eight-arm radial maze. However, in the study by Koubeissi et al. (2012), electrical stimulation seems to produce a more profound effect than chemical ablation. The patient was totally immobile and unresponsive. Our hypothesis (Smythies et al., 2012) might explain this in the following way. In the rats studied by Remedios (2012), their corticocortical pathway remains intact and thus could provide weak synchronization. However, 50 Hz electrical stimulation of the claustrum would disrupt not only intraclaustral synchronization, but might also traverse the claustrocortical projection and impact corticocortical synchronization, temporarily rendering the patient non-interactive with a profound lack of awareness – in essence, with “nothing.”

Thus, we suggest that a complex partial seizure results from a “blocked” claustrum, while a grand mal seizure is the result of a maximally activated claustrum.

## Code 2: Epigenetics

The claustrum is also notable on account of its susceptibility to kindling (Sheerin et al., 2004). Kindling is a phenomenon in which repeated short periods of electrical stimulation of a part of the brain results in a permanent lowering of its seizure threshold. The nature of the permanent, and therefore presumably structural, neuronal changes that bring about kindling are at present unknown. However, there could be such alterations at a number of different levels.

(1) There could be changes in such processes as the local molecular mechanisms that produce action potentials at the synapse, exert control of ion channels and uptake mechanisms, and alter of receptor processing by the endocytotic mechanisms.
(2) Structural changes in neurons can also be brought about by epigenetic mechanisms (Smythies and Edelstein, 2013).

There is now considerable data to show that plastic changes in the brain brought about by learning involve new protein synthesis, which is guided by epigenetic processes including DNA methylation and demethylation, as well as histone methylation and acetylation. Furthermore, a number of microRNAs can modulate the function and structure of neurons (Impey et al., 2009; Barmack et al., 2010; Cohen et al., 2011; Hsu et al., 2012; Saba et al., 2012). MicroRNAs bind to specific mRNAs and so control new protein synthesis at the mRNA level. Lastly, it has been shown that neuronal synaptic activity induces changes in all these epigenetic activities. This is relevant to the fact that kindling is induced by electrical stimulation. For example, Cohen et al. (2011) have identified a developmentally- and activity-regulated microRNA (miR-485) that controls dendritic spine number and synapse formation in an activity-dependent homeostatic manner. Transcription of the microRNA miP335 is promoted by naturally evoked synaptic activity at the climbing fiber-Purkinje cell synapse in the mouse cerebellar flocculus (Barmack et al., 2010). Impey et al. (2009) report that neuronal activity regulates spine formation, in part, by increasing miR132 transcription. MicroR-181a activity in primary neurons, induced by dopamine signaling, is a negative post-transcriptional regulator of GluA2 expression (Saba et al., 2012). Thus, it is possible that excess activity in kindled neurons could activate microRNAs by epigenetic mechanisms. This might induce the permanent effects reported.

In most instances these effects have been investigated in the same neuron that produced the epigenetic factors, or in neurons to which these agents were applied from an external source. However, there is developing evidence that transcription factors, mRNAs, and microRNAs can be transmitted from one neuron to another across the synaptic cleft. These are carried by exosomes. Exosomes are small lipoprotein vesicles, derived from multivesicular bodies and the cellular endosome system, that cross the external plasma membrane to enter the perisynaptic space (Fauré et al., 2006; Smalheiser, 2007; Lachenal et al., 2011; Chivet et al., 2012). Here, they gain entry to the postsynaptic neuron, most likely via astrocytes. The endosome membrane fuses with the external membrane of the postsynaptic cell, thus releasing its payload into the postsynaptic neuron. The payloads carried by these exosomes include epigenetic signaling molecules, such as signaling proteins and various types of RNA including mRNAs and microRNAs (Smalheiser, 2007; Lee et al., 2012; O'Loughlin et al., 2012; Tetta et al., 2012; Vlassov et al., 2012). From the host cytoplasm a system of carrier molecules could deliver the cargo widespread throughout the postsynaptic neuron. This new information has profound consequences for neuroscience. In this way the presynaptic neuron can modulate the functional structure of the postsynaptic neuron. It has been suggested that this system in the brain could be involved in information processing (Smalheiser, 2007; Chivet et al., 2012) and to form an epigenetic code (Smythies and Edelstein, 2013).

Thus, the brain may contain not one but two signaling systems. The first is the familiar one carried by the complex and exact timing of axon spikes and local potentials. This contributes to the computations the brain carries out and may be viewed as “software.” This may be involved in complex partial seizures and grand mal. The second signaling system may be an epigenetic code expressed by the precise quantities of signaling proteins, mRNAs and microRNAs carried along the axon of the presynaptic neuron, and transmitted across the synapse to modulate the structure and function of the postsynaptic neuron. This process structures the actual computational “hardware” in the neuron so as to enable it to optimally process the particular software it receives. In this way, the different modalities of cerebral cortex may have different computational hardware modulated by Code 2. This process could be involved in the genesis of kindling via the pathologically increased activation of neurons, resulting in the delivery of excessive amounts of epigenetic factors. This over-activity would translate into structural changes in the recipient neurons that would contribute to the neurological basis of the kindling effect.

The brain may be a computer, but it appears to be a most remarkable one in that it can alter its own hardware to fit changes in the software at hand.
